# Brief physical activity counselling by physiotherapists (BEHAVIOUR): protocol for an effectiveness-implementation hybrid type II cluster randomised controlled trial

**DOI:** 10.1186/s43058-022-00291-5

**Published:** 2022-04-08

**Authors:** Leanne Hassett, Matthew Jennings, Bernadette Brady, Marina Pinheiro, Abby Haynes, Balwinder Sidhu, Lauren Christie, Sarah Dennis, Alison Pearce, Kirsten Howard, Colin Greaves, Catherine Sherrington

**Affiliations:** 1 Institute for Musculoskeletal Health, The University of Sydney/Sydney Local Health District, Sydney, Australia; 2 Sydney School of Health Sciences, Faculty of Medicine and Health, University of Sydney, Sydney, Australia; 3 Liverpool Hospital, South Western Sydney Local Health District, Sydney, Australia; 4 Sydney School of Public Health, Faculty of Medicine and Health, The University of Sydney, Sydney, Australia; 5 Multicultural Health Unit, South Western Sydney Local Health District, Sydney, Australia; 6 St Vincent’s Health Network Sydney & Nursing Research Institute, Australian Catholic University, Sydney, Australia; 7 Ingham Institute, South Western Sydney Local Health District, Sydney, Australia; 8 University of Birmingham, Birmingham, UK

**Keywords:** Physical activity, Physical therapy, Counselling, Implementation, Healthcare, Behaviour change

## Abstract

**Background:**

Physical inactivity is a leading risk factor for chronic disease. Brief physical activity counselling delivered within healthcare systems has been shown to increase physical activity levels; however, implementation efforts have mostly targeted primary healthcare and uptake has been sub-optimal. The Brief Physical Activity Counselling by Physiotherapists (BEHAVIOUR) trial aims to address this evidence-practice gap by evaluating (i) the effectiveness of a multi-faceted implementation strategy, relative to usual practice for improving the proportion of patients receiving brief physical activity counselling as part of their routine hospital-based physiotherapy care and (ii) effectiveness of brief physical activity counselling embedded in routine physiotherapy care, relative to routine physiotherapy care, at improving physical activity levels among patients receiving physiotherapy care.

**Methods:**

Effectiveness-implementation hybrid type II cluster randomised controlled trial with embedded economic evaluation, qualitative study and culturally adapted patient-level outcome measures. The trial will be conducted across five hospitals in a local health district in Sydney, Australia, with a lower socioeconomic and culturally diverse population. The evidence-based intervention is brief physical activity counselling informed by the 5As counselling model and behavioural theory, embedded into routine physiotherapy care. The multi-faceted strategy to support the implementation of the counselling intervention was developed with key stakeholders guided by the Consolidated Framework for Implementation Research and the Capabilities, Opportunities, Motivation-Behaviour (COM-B) theoretical model, and consists of clinician education and training, creating a learning collaborative, tailored strategies to address community referral barriers, team facilitation, and audit and feedback. Thirty teams of physiotherapists will be randomised to receive the multi-faceted implementation strategy immediately or after a 9-month delay. Each physiotherapy team will recruit an average of 10 patients (*n*=300) to collect effectiveness outcomes at baseline and 6 months. The primary effectiveness outcome is self-reported planned physical activity using the Incidental and Planned Exercise Questionnaire, and the primary implementation outcome is reach (proportion of eligible new physiotherapy patients who receive brief physical activity counselling). Secondary effectiveness and implementation outcomes will also be collected.

**Discussion:**

This project focuses on physiotherapists as health professionals with the requisite skills and patterns of practice to tackle the increasing burden of chronic disease in a high-risk population.

**Trial registration:**

ANZCTR, ACTRN12621000194864. Registered on 23 February 2021.

**Supplementary Information:**

The online version contains supplementary material available at 10.1186/s43058-022-00291-5.

Contributions to the literature

- Physical inactivity is a leading risk factor for chronic disease and increases the economic burden on healthcare systems. Brief physical activity counselling embedded in healthcare can reduce physical inactivity, but implementation has been sub-optimal. This study addresses this important evidence-practice gap.
- This study will test both the implementation and effectiveness of brief physical activity counselling delivered by hospital-based physiotherapists to patients living in a lower socioeconomic and culturally diverse areas.
- This study will contribute to the recognised gap in the literature on diverse participant recruitment into trials by testing targeted approaches to recruit and retain patients from culturally and linguistically diverse populations.

## Background

Physical activity counselling refers to a component of patient consultation aimed at changing physical activity behaviour as a means of primary or secondary prevention of lifestyle-related chronic health conditions [1]. Physical activity counselling interventions that are underpinned by theoretical models of behaviour change and incorporate behaviour change techniques including identifying barriers, self-monitoring, goal setting and feedback have been shown to increase physical activity in the general population [2–4] as well as people with physical disabilities [5]. Brief physical activity counselling interventions such as the 5As approach (Assess, Advise, Agree, Assist and Arrange) [1] have shown to enhance physical activity with small to medium effects when delivered in healthcare settings by medical practitioners, practice nurses, physiotherapists and other healthcare professionals [6–8].

International [9, 10] and national [11] health organisations recommend that health professionals promote physical activity within their routine practice, and health professionals themselves strongly believe that they should be doing this [12, 13]. However, healthcare systems have failed to implement and scale-up physical activity promotion. Surveys of health professionals [12–15], audits of medical records [16] and surveys of patients [16–19] from around the world confirm that physical activity promotion, including counselling, is not routinely delivered in healthcare. Health professionals report that common barriers to delivery of this intervention include insufficient reimbursement, resources, time, knowledge, skills, training, protocols and organisational routines that do not support implementation [12, 13, 20, 21].

Physiotherapists are ideally placed to deliver physical activity counselling within their routine healthcare interactions. Physiotherapists can effectively deliver physical activity counselling [22], and patients see them as trustworthy physical activity messengers [23]. Physiotherapists have expertise in exercise prescription for a range of different health conditions and typically see patients on multiple occasions over an extended period which is ideal for supporting behaviour change [24]. Physiotherapists surveyed around the world agree that the promotion of physical activity should be part of their clinical role [25–28]. However, current efforts to put this into practice have been sub-optimal with only 36 to 54% of physiotherapists surveyed in different countries reporting they promote physical activity beyond therapeutic exercise to 10 or more patients per month [25, 26, 28]. Physiotherapists report similar barriers to implementation as other healthcare professionals [25–28].

The Brief Physical Activity Counselling by Physiotherapists (BEHAVIOUR) cluster randomised controlled trial will aim to address this evidence-practice gap by delivering a multi-faceted implementation strategy to teams of hospital-based physiotherapists to support them to implement brief physical activity counselling into routine care, which will, in turn, increase physical activity levels of patients receiving physiotherapy. A cluster design has been chosen to avoid the risk of contamination among individual physiotherapists working together within clinical teams. Although there is strong evidence for brief physical activity counselling in healthcare, few studies have been conducted with physiotherapists working in a public hospital setting, and few studies have been conducted in lower socioeconomic areas with patients from culturally and linguistically diverse backgrounds. Consequently, we cannot be certain that the implementation of brief physical activity counselling in this setting will increase the physical activity levels of the population of interest. Therefore, we will conduct an effectiveness-implementation hybrid type II study [29] with equal interest in implementation and effectiveness outcomes. This design is particularly suited to situations such as ours where there is good buy-in in the clinical setting, but intervention effectiveness is not yet known for this particular setting and population [30].

The co-primary effectiveness and implementation research questions are as follows:

1. Do patients receiving physiotherapy care participate in greater amounts of planned physical activity at 6 months if their physiotherapist received the multi-faceted implementation strategy to support the implementation of brief physical activity counselling in routine care, compared to patients whose physiotherapist did not receive the multi-faceted implementation strategy (primary effectiveness outcome)?
2. Do a greater proportion of patients receive brief physical activity counselling within routine physiotherapy care by physiotherapists who have received the multi-faceted implementation strategy compared to those who have not (reach, primary implementation outcome)?

Secondary research questions are as follows:

1. Do patients receiving physiotherapy care participate in greater amounts of overall physical activity (self-reported, device-based [sub-sample]), report greater function and quality of life, fewer barriers to physical activity and better impressions of physiotherapy care at 6 months if their physiotherapist received the multi-faceted implementation strategy to support the implementation of brief physical activity counselling in routine care, compared to patients whose physiotherapist did not receive the multi-faceted implementation strategy?
2. What is the adoption, reach, dose delivered, fidelity and sustainability of the delivery of brief physical activity counselling in routine physiotherapy care, and the delivery of the multi-faceted implementation strategy?
3. What are physiotherapists’ experiences and reported barriers and facilitators to engage with the multi-faceted implementation strategy and delivering physical activity counselling within routine physiotherapy care?
4. What is the cost-effectiveness of the multi-faceted implementation strategy and delivery of physical activity counselling in routine physiotherapy care?
5. How successful are the targeted trial participation approaches for optimising participation of patients from Culturally and Linguistically Diverse (CALD) backgrounds in the trial, in terms of reach and retention?

We hypothesise that physiotherapists who receive the multi-faceted implementation strategy will be better able and more motivated to incorporate brief physical activity counselling into routine care (measured by reach, dose delivered and fidelity of the physical activity counselling) than physiotherapists who have not received the multi-faceted implementation strategy. In turn, we hypothesise that patients who receive their physiotherapy care from a physiotherapist who has participated in the multi-faceted implementation strategy will be more likely to achieve greater amounts of planned physical activity at 6-month follow-up than patients who receive physiotherapy care from a physiotherapist who has not yet participated in the multi-faceted implementation strategy. We also hypothesise that the multi-faceted implementation strategy will be a cost-effective way to achieve practice change and greater physical activity among patients.

## Methods

### Design

**Fig. 1:**
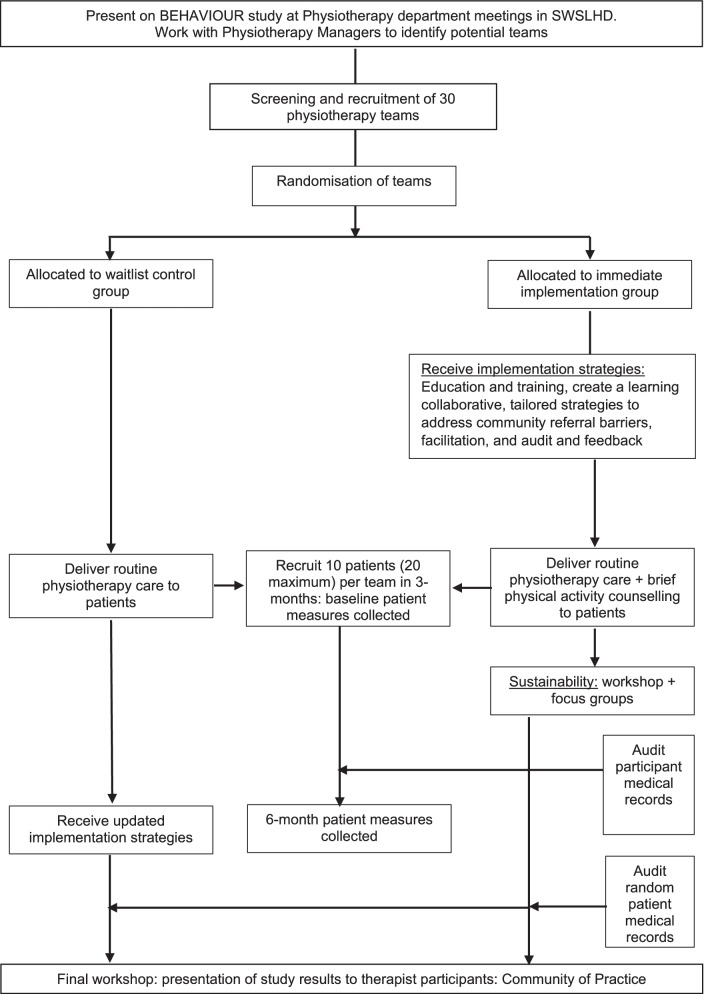
Flow diagram of study design

A cluster randomised controlled superiority trial with two parallel groups and 30 clusters will be undertaken. Qualitative and economic evaluation sub-studies, and cross-cultural adaptations of patient-level outcome measures will be embedded in the trial. The study will use an effectiveness-implementation hybrid type II design [29]. The trial design is presented in Fig. 1 and is guided by the Consolidated Standards of Reporting Trials (CONSORT): extension to cluster randomised controlled trials [31] and reported according to the Standard Protocol Items: Recommendations for Interventional Trials (SPIRIT) [32], the Standards for Reporting Implementation Studies (StaRI) Statement [33] and the TIDieR framework for evidence-based intervention description [34]. This study was approved by the South Western Sydney Local Health District Human Research Ethics Committee (2019/ETH13622; 2019/STE17765) and registered prospectively on the Australian and New Zealand Clinical Trials Registry (ACTRN12621000194864).

### Consumer and health professional input

This study was co-designed with our clinical investigators (MJ, BB, BS) and refined with local health professional input including physiotherapy managers and senior and junior physiotherapists via informal consultations and site visits, and formally via survey [24] as part of pre-implementation work led by LH. Consultation with staff from the District Multicultural Health Service informed our targeted approaches to include diverse participants and consumer representatives from the District and our research Institute Consumer panels provided input on patient-facing resources.

### Study sites and participant groups

This trial will be conducted across the South Western Sydney Local Health District (SWSLHD), a large health district within the state of New South Wales, Australia. This district includes lower socioeconomic areas, services almost one million people in both suburban and rural communities, 51% of households speak a language other than English at home and physical activity levels are 9% lower than the state average [35, 36]. The health district includes five public hospitals employing approximately 166 physiotherapists, and this study will recruit through physiotherapy departments at all five hospitals.

#### Therapist inclusion criteria

A hospital clinical team that provides a service for inpatients or outpatients within SWSLHD and has one or more physiotherapists within the team will form each of the 30 clusters. The size of clinical teams is likely to be different across different areas of physiotherapy and hospitals. Examples of clinical teams likely to be recruited include musculoskeletal outpatients, inpatient rehabilitation and pulmonary rehabilitation. For the clinical team to be included in the trial there needs to be at least one physiotherapist within the team interested in participating and who meets the following eligibility criteria: they anticipate they will remain employed within SWSLHD for the next 12 months, are either permanent or on ≥ 6-month rotation in the identified clinical team, would treat ≥ 10 new eligible adult patients within their clinical team over a 3-month period and are not working in a split role across two clinical teams that have both been identified as potential clusters for the trial to avoid potential contamination.

#### Patient inclusion criteria

Patients will be recruited by the participating physiotherapists in each of the 30 clusters. Patient participants are likely to be affected by common and/or burdensome conditions such as, but not limited to, osteoarthritis, lower limb amputations, chronic pain, stroke, brain injury, cardiac and pulmonary disease. Patients will be eligible if they are at least 18 years old, are new patients of one of the participating physiotherapists during the recruitment period, live in the community (as opposed to residential care), able to leave their home without physical assistance from another person but may use a walking aid or wheelchair (anticipated within 6 months of recruitment), are without major cognitive impairment (e.g., diagnosis of dementia), do not have any medical reasons for which interventions aimed at increasing physical activity would be contra-indicated and have sufficient language capabilities to respond to written questionnaires in English, Arabic or Vietnamese.

### Recruitment and allocation

#### Clinical teams

Thirty clinical teams will be recruited as clusters and randomised to either the immediate-implementation group (who will receive the multi-faceted implementation strategy immediately) or the waitlist control group (who will receive an updated version of the multi-faceted implementation strategy at the end of the trial). Eligible clinical teams will be identified by the Chief Investigator (LH) in collaboration with the District Physiotherapy Director (MJ) and the Physiotherapy Head of Department at each hospital site. The Chief Investigator will present the study protocol at department meetings and the Head of Department will then liaise with their staff regarding interest and eligibility to participate. Physiotherapists within eligible teams who agree to participate will be consented prior to the team being randomised. The randomisation schedule will be generated and managed by the Clinical Trials Centre at The University of Sydney who are not involved in the recruitment of clusters to ensure concealed allocation. The randomisation schedule includes minimisation based on the average number of occasions of service delivered by each clinical team (high dose vs. low dose i.e. equal to or above vs. below the median number of occasions of service) [24]. When there is a team(s) to randomise, the Chief Investigator will email the Clinical Trials Centre who will complete the randomisation and email the team allocation within 24 h.

#### Patient-participants

Consecutive new patients of participating physiotherapists within the 30 clinical teams who meet the eligibility criteria will be invited to participate in the study by their treating physiotherapist. Each eligible patient will be provided with a written participant information sheet and encouraged to discuss their participation with family. At their next physiotherapy session, the physiotherapist will consent the patient and provide them with a paper-based survey to complete. The patients will be blinded to the implementation aspect of the study and told the focus is to evaluate how people recover and get back to activity after receiving physiotherapy care.

### Evidence-based intervention

The evidence-based intervention to be implemented into practice is brief physical activity counselling embedded in routine care by hospital-based physiotherapists. The intervention development has been led by LH with oversight from CG who has extensive expertise in developing behaviour change interventions. The intervention is based on current evidence of effective elements of physical activity counselling [2, 4] and is informed by the 5As model of brief counselling (Assess, Advise, Agree, Assist, Arrange) [1] and behavioural theory [37, 38], with the use of motivational interviewing techniques and culturally appropriate language to ensure person-centred care. Table 1 provides details of the components of the intervention using the TIDieR checklist [34]. Any adaptations to the counselling intervention throughout the trial will be documented using the Framework for Reporting Adaptations and Modifications to evidence-based interventions [39].

**Table 1:** Evidence-based intervention description using the Template for Intervention Description and Replication (TIDieR) checklist

Brief name	Brief physical activity counselling within routine physiotherapy care
**Why**	Physical inactivity is a global health problem, with estimated 5.3 million deaths per year. Brief physical activity counselling (e.g. 5As model) [1] delivered by healthcare professionals as part of routine care is seen as opportunistic healthcare to address a public health problem. Evidence indicates that for every 12 sedentary people who receive physical activity counselling within healthcare at least one will increase activity to meet physical activity guidelines for health benefits [6]. As a public health intervention, this has the potential for community-based health benefits and healthcare cost savings. A theoretical basis combining Capability Opportunity Motivation-Behaviour change theory and Self Determination Theory informs intervention components and underpins patient materials.
**What procedures**	Patients receive their usual physiotherapy care to address the therapeutic reason that they have been referred for physiotherapy. Within this routine care, the physiotherapist will incorporate brief physical activity counselling comprising the following: ➢ Ask: Raise the topic of physical activity with permission (use motivational interviewing techniques and consider cultural influences throughout). ➢ Assess: Current physical activity level (objective and/or subjective); influences on physical activity using COM-B model. ➢ Advise: Discuss the benefits of physical activity; benefits of change; amount, type and intensity of physical activity (use elicit-provide-elicit framework). ➢ Agree: Collaboratively set 6–12-month behavioural sustainable goal(s); set short-term physical activity goals (use confidence scales for goal setting); develop action plan: what, where, when, how much, who with (include what if…. to anticipate barriers). ➢ Assist: Self-monitoring strategy (checklist, practice sheet, step count), assess/re-assess physical activity and goals, discuss barriers/facilitators, collaboratively identify community/home physical activity options, share plan for social support, build competence, control, connection (Self-Determination Theory). ➢ Arrange: Referral/contact/recommendation for healthcare/community/home-based physical activity, social support for practical (e.g. transport), emotional and motivational (e.g. family, friend, neighbour, carer, exercise/health professional)
**What materials**	Physiotherapists have access to a Microsoft Teams site that includes patient-facing resources: ➢ Handouts on physical activity guidelines and benefits of physical activity for a range of health conditions and produced by a number of organisations (e.g. Moving Medicine UK, Arthritis Association Australia, Australia National Disability Service Scheme, Cancer Council of Australia). Where available, handouts are available in English, Arabic and Vietnamese. ➢ Worksheets developed from a range of organisations (e.g. Agency for Clinical Innovation, National Institute on Aging) ➢ Study-developed worksheets including action plans, goal setting sheets, practice sheets, checklists, pedometer recording sheets, activity diary, Smartphone step counting instructions. ➢ Study-developed searchable activity directory with details of local physical activity opportunities that can be searched based on type of activity, cost, location. In addition, each team was provided with 10 pedometers (Yamax CW300) to use as they wished with patients.
**Who provided**	The intervention will be delivered by tertiary trained physiotherapists employed in the local health district who consent to participate in the study. Physiotherapists in the teams randomised to the immediate group, will receive the multi-faceted implementation strategy to support them to deliver physical activity counselling within routine care.
**How**	The physical activity counselling is delivered within routine care. How routine care is delivered may differ between teams and hospital sites but is most likely to be face-to-face (some virtual if COVID-19 restrictions apply). Any handouts, booklets or pedometers will be provided during the face-to-face session.
**Where**	Where the intervention is delivered may differ between teams and hospital sites but is likely to be delivered in an inpatient, outpatient or community setting within the boundaries of South Western Sydney Local Health District in the state of New South Wales, Australia.
**When and how much**	The frequency and duration of routine care will differ between services and sites and patient types. Data collected as part of pre-implementation work found that regardless of setting (inpatient, outpatient, mixed), physiotherapists reported an average of at least six occasions of service per patient lasting over 30 min per session [24]. Brief physical activity counselling is likely to be < 5min within these usual care sessions.
**Tailoring**	This is a tailored intervention. Depending on patient type, setting and frequency of interaction, the components of physical activity counselling will be incorporated. The core elements that all should receive include assess, advise and arrange using motivational interviewing techniques.

### Multi-faceted implementation strategy

The multi-faceted implementation strategy will be delivered by the research team to clinical physiotherapists randomised to the immediate-implementation group within their clinical teams (where possible) to support them to incorporate brief physical activity counselling into their routine practice. The primary implementation strategies will include education, training, tailored strategies to address community referral barriers, creation of a learning collaborative across the district, team facilitation, and audit and feedback to build clinicians’ Capabilities, Opportunities and Motivations to deliver brief physical activity counselling within routine care (see Table 2).

**Table 2:** Description of multi-faceted implementation strategy delivered to teams of physiotherapists mapped to Behaviour Change Wheel [37]

Implementation strategy [40]^**a**^	Mode of delivery/where/length/who delivered	Time-frame	Proposed mechanism of action			Intervention content	
			Barriers targeted	COM-B domains [41]	COM-B intervention functions	BCTs	Detailed explanation
Education	Series of short (2–8 min) education videos /online/~ 45 min in total/ LH, BB, research physiotherapists, consumers	Prior to attending workshop 1	Knowledge of PA guidelines, benefits of PA, consequences of inactivity, % meeting guidelines, what is PA counselling, what is the evidence for PA counselling and “what works” for supporting behaviour change, how many people to treat to have an effect, how many physiotherapists currently do it and if they don’t what stops them (include local data), what do patients think	Capability Psychological-knowledge; Motivation-reflective & automatic	Education; persuasion	Information about health consequences; Feedback on behaviour; Feedback on the outcome(s) of the behaviour; Information about others’ approval; Credible source; Salience of consequences; Feedback on behaviour; Social comparison; Identification of self as a role model	Short videos on the study, what is PA, what are the PA guidelines, benefits of PA from a patient perspective, what is PA counselling, behaviour change theories, raising the topic of PA as PT, raising the topic of PA with CALD patients
Education and training (conduct educational meetings and ongoing training, make training dynamic, promote adaptability)	Workshops 1 & 2: face-to-face workshop/at team’s hospital/maximum 4 h/LH	Workshop 1: 2–3 weeks after hospital rotation Workshop 2: 3 months later	How to do the different elements of PA counselling within usual care (Ask, Assess, Advise, Agree, Assist, Arrange, incorporating motivational interviewing techniques and behaviour change theories); skills at initiation & negotiating discussions about PA; prioritizing within usual care session; forgetting to ask or document about PA; lack of resources to do PA counselling; PA counselling not usual practice in clinical teams; building motivation in patients	Capability Psychological-cognition, interpersonal & self-regulation; Motivation-reflective & automatic; Opportunities-physical & social	Education; training; enablement; persuasion; modelling; environmental restructuring	Instruction on how to perform a behaviour; demonstration of the behaviour; feedback on the behaviour; behavioural practice/rehearsal; social support (practical); goal setting (behaviour); action planning; information about others’ approval; social comparison; identification of self as a role model; prompts/cues; adding objects to the environment; Problem-solving	Made up of presentations (live & pre-recorded) from PTs, behavioural expert, CALD expert, patients (e.g. how to do the different elements of PA counselling and resources to support this); tasks (e.g. role-playing, using scripts, reviewing resources), discussions (e.g. group sharing reflections & experiences of the content presented and practised). Paper manual with slides and resources given to each participant); link to online resources including https://movingmedicine.ac.uk/
Create a learning collaborative	Communication platform/online-Microsoft Teams/length project/MJ, LH	Added at workshop 1	Lack of resources to set action plans, find local opportunities, measure PA, PA counselling not usual practice in clinical teams, promoting PA counselling not seen as a priority across the district, lack of time to find resources	Opportunity-social & physical; Motivation-reflective	Training; enablement; modelling; persuasion	Adding objects to the environment; social support (practical); problem-solving; action planning; social support (emotional); feedback on the behaviour	Access to online videos and workshop presentations, sharing resources developed across teams, place to communicate, ask questions, share feedback on audit
Tailored strategies to address community referral barriers (capture and share local knowledge)	Not yet determined, may vary between teams/LH, the research team	Start at workshop 1	How to find PA opportunities in the local community, how to refer to local community PA opportunities	Capability Psychological-cognition & knowledge; Motivation-automatic; Opportunity-physical & social	Environmental restructuring; enablement; modelling; training	Adding objects to the environment; social support (practical); problem-solving; demonstration of the behaviour; instruction on how to perform a behaviour	Provide training in finding PA opportunities, help to develop links, referral resources, evaluation tools of community PA opportunities
Facilitation^b^ (change record systems, promote adaptability, capture and share local knowledge) and Audit & Feedback within teams	Mix face-to-face & remote/in their clinical settings/monthly (1–2h)/LH	Between workshops 1 and 2	Fitting PA in usual sessions including measuring PA; lack of suitable PA resources (for physiotherapist & patient); how to find PA opportunities in the local community; how to refer to local community PA opportunities; PA counselling not usual practice in clinical teams	Opportunity-physical & social; Capability-knowledge & cognition; Motivation-reflective & automatic	Education; training; enablement; modelling; environmental restructuring	Demonstration of the behaviour; social support (practical); self-monitoring of behaviour; problem-solving; action planning; social support (emotional); feedback on the behaviour; behavioural practice/rehearsal; prompts/cues; adding objects to the environment	Working with the team to identify/modify/develop resources for their team (including local activity directory or similar resource), identify & connect with appropriate PA opportunities, modify physiotherapy assessment forms to include PA information to collect, audit and feedback with a colleague between workshops.

Key: *PA* Physical activity, *CALD* Culturally And Linguistically Diverse, *PT* Physiotherapist, *BCTs* Behaviour Change Techniques

^a^Secondary implementation strategies are in brackets; ^b^Facilitation: “A process of interactive problem solving and support that occurs in a context of a recognized need for improvement and a supportive interpersonal relationship” p.9 [40]

The development and delivery of the multi-faceted implementation strategy is led by LH (with input from CG and BB for the education and training aspects) who has a 17-year experience as a clinical physiotherapist, has had training in behaviour change methodology, implementation science and motivational interviewing, and holds an honorary position in the local health district. The Consolidated Framework for Implementation Research (CFIR) [42] has been used as an overarching framework to guide the multi-faceted implementation strategy development (see Supplemental file 1). The implementation strategies were derived from the Expert Recommendations for Implementing Change (ERIC) project [40] and the Behaviour Change Wheel [37] and are informed by literature describing barriers that physiotherapists report stop them from incorporating brief physical activity counselling into routine practice [25–28] as well as local barriers identified during informal consultations and a formal survey with clinical physiotherapists in the district as part of pre-implementation work [24]. Any adaptations to the implementation strategies will be documented throughout the trial using the Framework for Reporting Adaptations and Modifications to implementation strategies [43].

### Data collection methods and outcome measures

As a hybrid type II study [29], patient-level effectiveness and implementation outcomes will be measured. All effectiveness outcomes (excluding device-measured physical activity and impressions of physiotherapy) as well as demographic information and co-morbidities will be assessed at baseline to enable a description of the patient-participant’s baseline characteristics and to obtain values to enter as covariates in the models comparing groups at follow-up. At the time of consent, the treating physiotherapist will provide the participant with a copy of the paper-based survey. This will be completed independently by participants or assisted by study staff over the telephone who are blinded to physiotherapy team allocation. At 6 months post-baseline, follow-up paper-based surveys will be mailed to participants and followed up by blinded research staff if surveys are not returned by mail.

#### Primary effectiveness outcome

Self-reported time (hours/week) spent in planned physical activity over the last 7 days at 6 months from baseline. This will be measured using the planned activity subscale of the Incidental and Planned Exercise Questionnaire (IPEQ). The self-report IPEQ has excellent psychometric properties and assesses the level of physical activity relating to both basic and more demanding activities [44]. Planned physical activity includes total time spent participating in exercise classes, home exercise, other sport or physical recreation activities, and walking for exercise.

#### Primary implementation outcome

The primary implementation outcome is reach, defined as the proportion of eligible new physiotherapy patients who receive brief physical activity counselling within routine physiotherapy care, measured from a checklist completed by the physiotherapists.

#### Secondary effectiveness outcomes

##### IPEQ_total

The total physical activity (planned and incidental) will be measured using the total time in hours/week self-reported using the IPEQ. The total physical activity includes the total time spent participating in planned physical activity as well as other walks, outside gardening and maintenance and time on feet indoors.

##### Average step count per day

This is measured using Actigraph accelerometer model GT3X *(Pensacola FL USA).* The Actigraph GT3X is a non-invasive tri-axial accelerometer. Participants will be instructed to wear the accelerometer for 7 consecutive days during waking hours (except during water activities or bathing). Daily step count will be averaged over valid weartime days (≥10 h per day) to give average steps per day as the variable of interest. This measure will only be collected for a sub-sample of the patient participants due to resources and as a way of testing the feasibility of device-based physical activity measurement for future scale-up studies.

##### Patient-Reported Outcomes Measurement Information System (PROMIS-29 v2)

The PROMIS-29+2 Profile v2.1 will be used to assess the physical functioning and mental wellbeing of participants. The PROMIS-29 includes a numeric rating (0 to 10) pain intensity scale and seven health domains (physical function, anxiety, depression, fatigue, sleep disturbance, participation in social roles and activities, and pain interference) each rated on a 5-point scale. Additionally, participants will be asked to answer two items from Cognitive Function – Abilities (PC6r and PC27r), so preference-based scores can be calculated [45, 46]. Seven domain scores, a pain intensity score and physical and mental health summary scores will be generated (raw and T-score) with preliminary evidence supporting the reliability and validity of this tool [47].

##### EuroQOL-5D (EQ5D-5L)

Utility-based quality of life will be measured using the EQ5D-5L, a 5-item quality of life scale measuring the domains of mobility, self-care, usual activities, pain or discomfort and anxiety or depression, each rated on a 5-point scale ranging from no problems with that domain to extreme problems or unable to do that domain. In addition, there is a visual analogue scale from 0 to 100 to indicate their current health status, with zero being the worst imaginable health status and 100 being the best imaginable health status. The EQ5D-5L provides five domain scores, visual analogue scale score (0 to 100) and overall health utility score [48, 49].

##### Barriers to Physical Activity Questionnaire for People with Mobility Impairments (BPAQ-MI)

Barriers to participation in physical activity will be measured using the BPAQ-MI [50]. This scale measures barriers to physical activity across the intrapersonal, interpersonal, organisational, and community domains and has shown good test-retest reliability and construct validity [50]. This scale lists 63 potential barriers within eight domains (health, beliefs/attitudes towards physical activity, friends, family, facility-built environment, staff/program/policy, community-built environment and safety) for the responder to identify barriers limiting their participation in physical activity. Any reported barrier is rated on a 5-point scale as to how limiting it is. Each barrier has an item weighting which is multiplied by the score for that barrier between 0 and 5. The eight domains and total scores are calculated.

##### Healthcare evaluation

A study-specific questionnaire will collect information on the participant’s impressions of their health care. Specifically, it will ask if they received advice about physical activity from their physiotherapist or other healthcare providers and if they experienced any adverse events while participating in physical activity over the last 6 months.

##### Healthcare utilisation

This will be collected within New South Wales Health directly from the Activity-Based Management portal. The frequency of all health encounters captured by this system (i.e., hospitalisations, emergency department presentations and non-admitted activities such as specialist consultation and physiotherapy) that participants from both groups have during the 6-month trial period will be collected and costed using routine sources including DRG-based costs from the National Hospital Cost Data Collection [51] for inpatient presentations and Medical Benefits Schedule (MBS) and Pharmaceutical Benefits Schedule (PBS) for outpatient utilisation to inform the trial-based economic evaluation.

#### Secondary implementation outcomes

Secondary implementation outcomes are measures of adoption, dose delivered, reach, fidelity and sustainability. These are measured both with respect to the delivery of brief physical activity counselling within routine physiotherapy care and delivery of the multi-faceted implementation strategy to support and train physiotherapists. These five measures are recommended as essential outcome measures in evaluating implementation studies [52]. See Table 3 for how each measure is being operationalised in this trial.

**Table 3:** List of implementation outcome measures included as part of the BEHAVIOUR study

Implementation outcomes	Delivery of physical activity counselling		Delivery of multi-faceted implementation strategy	
	Definition of outcome	Measurement tool	Definition of outcome	Measurement tool
Reach	Reach of the delivery of physical activity counselling will be measured in three ways: (1) Proportion of eligible new physiotherapy patients who receive physical activity counselling^a^; (2) Percentage of patient participants that are from culturally and linguistically diverse backgrounds; (3) Percentage of community physical activity providers referred to from number identified within the study.	1. Study screening log^b^ 2. Audit of baseline data records 3. Audit of study developed activity directory and physiotherapy medical notes.	The reach of the delivery of the multi-faceted implementation strategy will be measured in two ways: (1) the Proportion of the different types of clinical physiotherapy teams across the district that participate in the implementation strategies; (2) the Number of new community physical activity providers identified and added into the study-specific activity directory.	1. Study recruitment log; 2. Audit of activity directory
Adoption	The percentage and representativeness of providers (clinical teams) that will adopt physical activity counselling, will be measured by (1) The number of clinical teams across the district who participated compared to the number invited to participate; (2) Percentage of eligible physiotherapists within clinical teams who participated in the study (participated in implementation strategies and attempted to deliver physical activity counselling to their patients).	1. Audit of study records kept by research team 2. Study-specific training and resources log	Number of physiotherapy clinicians or managers across the district that contribute content, resources or teaching within the multi-facted implementation strategy.	Study-specific training and resources log
Dose delivered	The dose of physical activity counselling will be measured across immediate and waitlist groups by physiotherapist self-reported proportion of time spent on physical activity counselling within total usual care sessions for recruited patient participants in the trial.	Study screening log and hospital occasion of service statistics^b^	Average dose (hours) per clinical team of implementation strategies delivered (education and training, audit and feedback, facilitation sessions, learning collaborative, tailored strategies for community referral).	Study-specific training and resources log
Fidelity (adherence)	The fidelity of physical activity counselling (elements included) delivered to patient-participants will be measured across immediate and waitlist groups in two ways, self-report and audit.	1. Physiotherapist self-report study checklist^b^ 2. Audit of EMR of all patient-participants^b^	Fidelity of multi-facted implementation strategy delivered will be measured by the percentage of implementation strategies that are implemented as prescribed in the study protocol including modes of delivery; COM-B domains, categories and intervention functions; proposed content and behaviour change techniques.	Study-specific checklist
Sustainability (maintenance)	The sustainability of physical activity counselling (elements included) will be measured in intervention clinical teams in a random sample of patients (5 per team) after the patient recruitment period.	Audit of EMR	Percentage of clinical physiotherapists from immediate implementation group involved in delivering implementation strategies or providing resources to waitlist implementation group.	Study-specific training and resources log

*EMR* Electronic medical record

^a^Primary implementation outcome; ^b^Data collected for immediate and waitlist groups and between-group difference calculated

### Implementation determinants

**Fig. 2:**
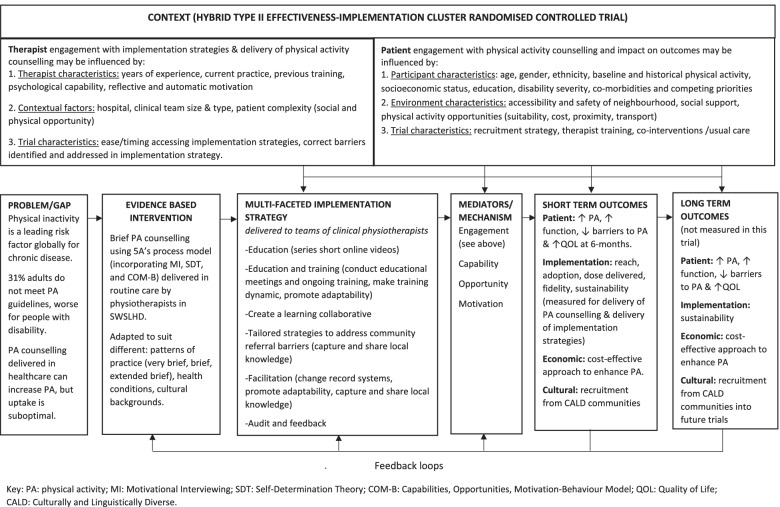
Logic model for the BEHAVIOUR trial

Implementation determinants will be evaluated using both quantitative and qualitative research methods. Patient-participant and physiotherapist factors (collected from demographic information) as well as contextual factors (hospital, team, patient environment) and trial characteristics will be considered in the determination of implementation success. Change in clinician self-reported capabilities, opportunities and motivations after receiving the multi-faceted implementation strategy will be evaluated using a survey with five-point Likert scales, with one being strongly disagree and five being strongly agree. This survey is based on the COM-B self-evaluation questionnaire [41] and adapted from Webb et al. [53] (see Supplemental file 2). The focus groups will be conducted with physiotherapists who participated in the implementation strategies at the end of the patient recruitment phase, led by LC and overseen by AH, SD and BB. Normalization Process Theory [54], the CFIR [42] and the Consolidated Criteria for Reporting Qualitative Research (COREQ) [55] will guide the conduct and reporting of this qualitative study. Figure 2 presents the logic model for how these determinants are hypothesised to influence the outcomes of the trial.

### Strategies to include Culturally And Linguistically Diverse (CALD) patient-participants

Targeted approaches have been included within this study to include patients from Arabic and Vietnamese cultures, the two most prevalent CALD communities in South Western Sydney. Effectiveness outcome measures will be provided to the patient in their preferred language of English, Arabic or Vietnamese. Arabic and Vietnamese translations of the EQ5D-5L are available and have undergone testing of their psychometric properties [56, 57], and we received permission to use the following self-complete versions: Egypt, Arabic (version 1.1); Vietnam, Vietnamese (version 1.1) and Australian, English (version 1.1). The PROMIS-29 is available in English and Arabic, and we received permission to use the Arabic version. Within this project and led by BB, the PROMIS-29 was translated into Vietnamese using cross-cultural adaptations and forward and backward translation as per PROMIS-29 translation requirements. Following similar forward and backward translation processes, the IPEQ was also translated into Arabic and Vietnamese (processes to be reported in a separate publication). Finally, the other patient data collection forms were translated to Arabic and Vietnamese using a forward translation process and cross-checked by a team of multicultural health officers for accuracy.

To support the consent process, participant information sheets and consent forms have been translated into Arabic and Vietnamese. To further support informed consent, videos were developed in English, Arabic and Vietnamese by investigators BB and BS, in consultation with the SWSLHD Multicultural Health Service and consumer representatives from the Consumer and Community Participation Committee. The videos explain research concepts, reasons for participating in research and an overview of the study requirements in lay language, adapted for each target community. Access to these videos will be provided via a QR code attached to the participant information sheet on invitation to the study. To support the recruitment and retainment of CALD participants, the district Multicultural Health Service will provide support led by BS. Arabic and Vietnamese speaking multicultural health workers will provide telephone support at initial recruitment, assistance with the completion of survey questions at baseline and 6 months as required, and an additional check-in phone call at 3 months to support engagement with the project. These staff are blinded to physiotherapy team allocation.

### Data analysis and management

#### Sample size

The sample size calculation was undertaken in STATA 14.2 using the “clustersampsi” command. Recruitment of 300 patients undergoing physiotherapy is sufficient to detect a 1.5-h between-group difference in the self-reported time spent in planned physical activity per week, estimated at 3 h/week (SD 4.4 hours/week) (waitlist control group) to 4.5 h/week (immediate-implementation group) (a between-group difference found to be achievable in our previous study [58]). All calculations used 80% power, alpha = 0.025 (to account for co-primary outcomes), 15 clusters per arm, average cluster sizes of 10, baseline-covariance 0.6 and intracluster correlation 0.05. This sample size will also be sufficient to detect an absolute 25% difference in the proportion of eligible patients receiving brief physical activity counselling within routine physiotherapy care (primary implementation outcome (reach), 35% in the waitlist control group and 60% in the immediate-implementation group).

#### Data management

Patient screening and recruitment will be collected on encrypted Excel spreadsheets for each physiotherapy team, saved on the health service password-protected drive and shared securely with the research team. All paper-based participant data will be coded with a unique study ID number and will be entered into a password-protected REDCap database [59, 60] with a license held by The University of Sydney. Access to this database will only include the Chief Investigator and required members of the research team.

#### Quantitative analysis

The planned quantitative analysis has been designed by CS and LH, overseen initially by a local statistician. The analysis will be conducted by LH and CS using STATA statistical software. Primary analyses will be pre-planned and conducted while masked to group allocation. To account for correlation among individuals within clusters, all statistical models will use a generalised estimating equations (GEE) approach with an exchangeable correlation structure.

##### Patient-level effectiveness outcomes

The primary analysis will evaluate the effect of the multi-faceted implementation strategy to train, support and facilitate physiotherapists to deliver brief physical activity counselling as part of their routine care [targeted at the cluster level] on primary and secondary effectiveness outcomes. The main (intention-to-treat (ITT)) dataset analysed will be constituted of all patients recruited into the study irrespective of what amount of physical activity counselling they received from their treating physiotherapist. For the continuous outcomes, multiple linear regression will be used with a group (immediate-implementation vs. waitlist control) as the primary independent variable and baseline scores as a covariate. Binary logistic regression will be used to compare groups on dichotomous outcome measures with the group as the primary independent variable.

##### Implementation outcomes

Implementation outcomes are measured both with respect to the delivery of brief physical activity counselling within routine physiotherapy care and delivery of the multi-faceted implementation strategy to support and train physiotherapists. Some of these are measured for both groups with between-group statistical comparisons conducted, and others are measured only for the immediate-implementation group and the statistics will be presented descriptively (see Table 3). For the continuous outcomes measured for both groups evaluating the delivery of brief physical activity counselling (reach, dose delivered and fidelity), multiple linear regression will be used with the group as the primary independent variable. All other implementation outcomes will be descriptively presented.

#### Exploratory subgroup analysis

The study is not powered to detect between-group differences for subgroup analysis. However, exploratory and pre-specified subgroup analyses will be undertaken on the co-primary outcomes to help understand if these variables are likely to impact on the outcomes and should be included in future implementation trial designs. All subgroups will be defined by data collected prior to randomisation. Subgroup analysis will be conducted for patient gender (male versus female), age (entered as continuous and categorical (above and below median age) covariates), language spoken at home (English versus non-English), recruited from inpatient vs. outpatient physiotherapy team and recruited from high-dose vs. low-dose physiotherapy team.

#### Implementation determinants

Quantitative and qualitative data will be synthesised following mixed methods approaches [61] to understand implementation determinants. Change in physiotherapists’ self-reported Capabilities, Opportunities and Motivations to deliver physical activity counselling after receiving the implementation strategies will be presented as mean (SD) for each item. Univariate correlations will explore change in Capabilities, Opportunities and Motivations and Engagement (see Fig. 2) with the reach, dose delivered and fidelity of physical activity counselling provided to help understand key elements of implementation strategies for changing the behaviour of physiotherapists.

Audio-recordings of each post-implementation focus group or interview will be transcribed verbatim and imported into NVivo 12 (QSR International, Australia). A thematic analysis will be conducted to explore determinants of implementation outcomes. The coding frame will be informed by the CFIR [42] and Normalization Process Theory, supplemented by inductive coding to capture additional relevant information.

#### Economic analysis

The economic evaluation will be conducted by MP and overseen by KH and AP and will include a cost-effectiveness and cost-utility analysis and take a health care payer perspective, with the time horizon limited to the trial duration. Costs will include program delivery costs (including staff and equipment costs) and health service utilisation costs. The brief physical activity intervention delivery costs (physiotherapy staff hours and equipment costs) will be estimated using trial-specific data and costed using appropriate wage rates and on-costs. The multi-faceted implementation strategy preparation and delivery costs (research staff hours and resource development costs) will be estimated using trial-specific data and costed using appropriate wage rates and on-costs. Using the mean costs and the mean health outcomes in each trial arm, the incremental cost per (1) additional person achieving a clinically meaningful increase in physical activity based on the IPEQ, (2) additional person with any improvement in quality of life (from PROMIS-29) and (3) quality-adjusted life-year (QALY) gained (from EQ5D-5L) in the intervention group compared with the control group will be calculated. Bootstrapping will be used to estimate a distribution around costs and health outcomes and to calculate the confidence intervals around the incremental cost-effectiveness ratios (ICER). Results will be plotted on a cost-effectiveness plane. A range of additional sensitivity analysis will be conducted to determine the impact on the ICER of various assumptions within the economic evaluation, such as the efficacy or fidelity of the intervention in the longer term, strategies for handling missing data, use of alternative methods for QALY estimation, or different cost scenarios for alternative implementation strategies. A specific scenario analysis will also be conducted to evaluate the cost-effectiveness of multi-faceted implementation strategies and sustainability strategies being conducted by staff employed through the local health district rather than the research team.

## Discussion

The study is testing a potentially efficient model of care to reduce chronic disease by utilising the current workforce and embedding brief physical activity counselling into routine care. There is strong evidence to support this intervention and the World Health Organization’s Global Action Plan on Physical Activity 2018–2030 supports this strategy as one of its’ 20 policy actions to achieve a 15% relative reduction in the global prevalence of physical inactivity by 2030 [10]. The conduct of a district-wide implementation project also has potential additional benefits above the impact on physical inactivity. It is also promoting the local health district as a “learning health system” [30] to ensure high-value care and embedding research as part of clinical practice. A potential risk is additional demands on clinicians’ time; however, the multi-faceted implementation strategy has been designed with key local stakeholder input to ensure the strategies fit within current systems.

A further potential risk is contamination between physiotherapists in intervention and control clusters. This risk has been considered carefully when designing the study. A cluster trial as opposed to an individually randomised controlled trial has been selected to reduce this risk and the trial start date has been timed with the beginning of staff rotations. It is still likely that some staff will change and rotate out of clinical teams due to unforeseen circumstances. In this instance, the physiotherapist will still be welcome to participate in the implementation strategies; however, they will not recruit any patients into the trial in their new clinical team and will be asked not to share any training resources within their new team. An existing or new member of that team not currently involved in the trial may be invited to join the study within that cluster. A final risk is the impact of COVID-19 on clinical team makeup, how routine care is delivered and opportunities for community-based physical activity for patients. The impact of COVID-19 will be carefully documented, and we will work closely with our clinical investigators to navigate this risk.

The benefits of physical activity have been well established for many years, yet the predominant research studies published are still observational studies with only 16.7% categorised as implementation and scale-up studies [62]. By using the hybrid type II design, our study will provide essential information about how this intervention can be adopted and delivered in a real-world setting while simultaneously evaluating the effectiveness of the intervention in these real-world conditions. This will provide direct evidence to policy makers as to if and how the intervention can be implemented and scaled up at a systems level to meet population health goals [62].

## Supplementary Information

**Additional file 1: Supplemental file 1.** Use of Consolidated Framework for Implementation Research (CFIR) as an overarching implementation framework for the BEHAVIOUR trial. **Supplemental file 2.** Determinants of implementation questionnaire.

## Data Availability

De-identified patient-level data for primary and secondary outcomes will be made available when the main study results are published and will be available through the University of Sydney’s open access institutional repository (htts://ses.library.usyd.edu.au/).

## Abbreviations

BEHAVIOUR: Brief Physical Activity by Physiotherapists
COM-B: Capability, Opportunity, Motivation- Behaviour theory of behaviour change
5As: Assess, Advise, Agree, Assist, Arrange
CALD: Culturally And Linguistically Diverse
SWSLHD: South Western Sydney Local Health District
CFIR: Consolidated Framework for Implementation Research
IPEQ: Incidental and Planned Exercise Questionnaire
PROMIS-29: Patient-Reported Outcomes Measurement Information System
EQ5D-5L: EuroQOL-5D
BPAQ-MI: Barriers to Physical Activity Questionnaire for People with Mobility Impairments
ICER: Incremental Cost-Effectiveness Ratios
QALY: Quality Adjusted Life Year
