# Supplementary material for: Brief physical activity counselling by physiotherapists (BEHAVIOUR): protocol for an effectiveness-implementation hybrid type II cluster randomised controlled trial
Source: Implement Sci Commun. 2022 Apr 8;3:39. doi: 10.1186/s43058-022-00291-5 (PMC8991667; doi:10.1186/s43058-022-00291-5)
Supplement: Supplementary file 1 — Additional file 1: Supplemental file 1. Use of Consolidated Framework for Implementation Research (CFIR) as an overarching implementation framework for the BEHAVIOUR trial. Supplemental file 2. Determinants of implementation questionnaire. [file 43058_2022_291_MOESM1_ESM.docx]

**Supplementary Files**

1. Overarching implementation framework for BEHAVIOUR: Consolidated Implementation Research Framework (CFIR).
2. Determinants of implementation questionnaire.

**Supplemental File 1:** Use of Consolidated Framework for Implementation Research (CFIR) as an overarching implementation framework for the BEHAVIOUR trial.

| **Constructs** | **Relevance/inclusion in BEHAVIOUR** | **Measurement** |
| --- | --- | --- |
| **I. INTERVENTION CHARACTERISTICS** | | |
| 1. Intervention source /development | Project externally initiated with close collaboration on development internally (Physiotherapy district Director). Further internal development with clinical specialist physiotherapist, A/Prof Allied Health, discussions with Physiotherapy Head of Departments (HODs), clinical physiotherapists. | Nil. |
| 1. Evidence Strength & Quality | Physiotherapists’ beliefs around quality of evidence for physical activity counselling assessed pre-implementation. Evidence of effectiveness will be presented in education and training workshops if indicated from behavioural diagnosis. Change in physiotherapists beliefs assessed post workshops. | -Behavioural Diagnosis questionnaire^1^  -Implementation questionnaire |
| 1. Relative advantage | Physiotherapists’ beliefs around importance of physical activity counselling compared to other interventions assessed pre-implementation. | -Behavioural Diagnosis questionnaire^1^ |
| 1. Adaptability | Intervention to be presented as adaptable for different health conditions, different physiotherapy patterns of practice, and different cultural backgrounds (particularly Arabic and Vietnamese). | -Different number resources developed & used  -Explored in post-implementation focus groups with clinicians |
| 1. Trialability | Cluster RCT with waitlist-control group. Waitlist teams will receive updated version of implementation strategies with any changes to the counselling intervention dependent on feedback/measures from initial implementation. | -Number of changes/ additions to the counselling intervention. |
| 1. Complexity | Implementation strategy aimed to reduce intervention complexity (e.g., patient resources, scripts, information on local community opportunities). | -Explored in post-implementation focus groups with clinicians |
| 1. Design Quality and Packaging | Already developed professional resources used where appropriate, inhouse resources developed where necessary. | -Uptake of already developed and new inhouse resources |
| 1. Cost | Health economic analysis looking at cost of intervention and implementation strategy delivery. | -Health economic analysis |
| **II.** **OUTER SETTING** | | |
| A. Patient needs and resources | Evidence to be presented in workshops that patients want physiotherapists to discuss physical activity. Consumer input into patient-facing materials. Working with district multicultural health unit to meet needs Arabic and Vietnamese speaking patients. | -Percentage CALD patients recruited and retained in trial.  -Amount of consumer input on patient-facing materials |
| B. Cosmopolitanism | Plan to collect data on current physiotherapy links to community physical activity organisations. Working with clinicians as part of implementation strategy to increase links with local community opportunities. | -Percentage of appropriate community physical activity providers identified.  -Percentage patient referrals to community physical activity providers during trial. |
| C. Peer Pressure | Questions included in pre-implementation behavioural diagnosis regarding priority of providing physical activity advice from team, hospital, local health district and the physiotherapy profession perspectives. | -Behavioural Diagnosis questionnaire^1^ |
| D. External Policy & Incentives | International health guidelines as well as those from Australia recommend that every healthcare interaction by any health professional should be seen as an opportunity to promote physical activity. | Nil |
| **III.** **INNER SETTING** | | |
| A. Structural Characteristics | SWSLHD: Five public hospitals, estimated to service population 1 million, lower socioeconomic area, more than 50% households speak a language other than English. One of largest health districts in New South Wales. Range of experience in clinicians and managers. Stable senior management. | Nil |
| B. Networks & Communications | Within hospital: Physiotherapy Department led by Head of Department, with clinical areas led by team leaders and clinical teams led by senior physiotherapists. Across the district there is a Physiotherapy District Advisor and there are clinical network teams for key areas of physiotherapy. | -Explored in post-implementation focus groups with clinicians |
| C. Culture | Questions in pre-implementation behavioural diagnosis regarding social opportunity. As physiotherapists work in clinical teams, a cluster trial design is most appropriate to implement a new intervention within clinical teams. | -Behavioural Diagnosis questionnaires^1^ |
| D. Implementation Climate  1. Tension for Change  2. Compatibility  3. Relative Priority  4. Organizational Incentives & Rewards  5. Goals and Feedback  6. Learning Climate | District Advisor and HOD support for project. Input from HODs on mode of implementation delivery (e.g., workshop frequency, length). Timing of project to commence to match change of staff (new first year grads) and rotations. Start in Summer/Autumn as can get harder in Winter to prioritise additional activities with extra demands on hospital beds.  Clinicians were invited to indicate their interest to participate in the cluster trial, so we are starting with the most receptive clinicians. Potential for participating clinicians to become Clinical Champions of this intervention.  Pre-implementation behavioural diagnosis questionnaire includes questions on beliefs of importance of physical activity counselling and role of physiotherapists. Time for reflective thinking and evaluation built into implementation strategy. A challenge will be the crowded research/quality improvement/clinical initiatives meaning clinicians being asked to be involved in multiple projects and risk contamination to study when some projects overlap. | -Behavioural Diagnosis questionnaire^1^  - Explored in post-implementation focus groups with clinicians |
| E. Readiness for implementation  1. Leadership engagement  2. Available resources  3. Access to knowledge & information | Strong commitment of Physiotherapy Management team for this project. Head of Departments committed to clinician time away from clinical duties to participate in project. Free training facilities located. Grant applications supported by team to increase resources for project including grant application and additional funds commitment to better engage CALD population in trial. Resources developed in trial to be made freely available to clinicians in SWSLHD at the end of the trial. | - Explored in post-implementation focus groups with managers |
| **IV. CHARACTERISTICS OF INDIVIDUALS** | | |
| 1. Knowledge & Beliefs about the intervention | Pre-implementation behavioural diagnosis questionnaire includes questions about knowledge and beliefs of physical activity counselling. Will be included in implementation strategy if found to be a significant barrier. Post implementation questionnaire includes questions about whether knowledge and beliefs have changed. | -Behavioural Diagnosis questionnaire^1^  -Implementation questionnaire |
| 1. Self-efficacy | Pre-implementation behavioural diagnosis questionnaire includes questions about psychological capability and beliefs and feelings about their capabilities to deliver physical activity counselling. Post-implementation questionnaire evaluates change in this. | -Behavioural Diagnosis questionnaire^1^  -Implementation questionnaire |
| 1. Individual Stages of Change | Pre-implementation behavioural diagnosis questionnaire includes evaluation of current physical activity counselling practice. We may be able to categorise people into stages of change from this data. | -Behavioural Diagnosis questionnaire^1^ |
| 1. Individual Identification with Organization | Pre-implementation behavioural diagnosis questionnaire includes information on years working in SWSLHD and years working as a physiotherapist. | -Behavioural Diagnosis questionnaire^1^  - Explored in post-implementation focus groups with clinicians |
| 1. Other personal attributes | Demographic information collected as part of pre-implementation behavioural diagnosis questionnaire. Nil other personal attributes included at this time. | Nil. |
| **V. PROCESS** | | |
| A. Planning | Detailed planning process with input from local stakeholders, research team, evidence on physical activity counselling, evidence on implementation science, data from local context on evidence-practice gap, use of theoretical model of behaviour change (COM-B) and training in this model. Designed for local context and with scale-up in mind. | Nil. |
| B. Engaging  1. Opinion Leaders  2. Formally appointed internal implementation leaders  3. Champions  4. External Change Agents | Strategies for initial engagement have included: presentations to clinicians and managers in SWSLHD regarding physical activity in different forums (allied health research day, district research day, physiotherapy department meetings, district rehabilitation clinical network, team meetings, district Head of Department meetings), individual conversations with clinicians (including opinion leaders). Lead investigator is an honorary research fellow in the district (previous 17 years clinical work in district)- based in district 1 day per week. Key opinion leader clinical physiotherapist part of research team (and with CALD experience). Clinical physiotherapists engaged in project through invitation to complete Behavioural Diagnosis questionnaire within hospital department meeting. Identify clinical champions at different sites during implementation phase- invite involvement in delivering the implementation strategy to the waitlist control group. Potential outcome of project: formally appointed internal implementation leaders. | -Behavioural Diagnosis questionnaire^1^  -Economic evaluation cost effectiveness of delivery of implementation strategy |
| C. Executing | Evaluate whether planned aspects of implementation strategy are delivered. | Delivery of implementation strategy as planned evaluated with dose delivered and fidelity implementation outcome measures |
| D. Reflecting and Evaluating | Incorporated in implementation strategy and evaluation process. Workshop 2 focused on debriefing/sharing experiences of trying to incorporate physical activity counselling into practice, further training/support in areas required. Focus groups debrief/sharing experiences incorporating physical activity counselling into practice, establishing a community of practice. | -Implementation questionnaire  - Explored in post-implementation focus groups with clinicians |

^1^ Zhu S, Sherrington C, Jennings M, Brady B, Pinheiro M, Dennis S, et al. Current Practice of Physical Activity Counselling within Physiotherapy Usual Care and Influences on Its Use: A Cross-Sectional Survey. Int J Environ Res Public Health. 2021;18(9).

**Supplemental File 2** Determinants of implementation questionnaire

Post workshop questionnaire.

**Following today’s session please rate on a scale of 1 to 5 your agreement with the following statements with**

**1. being strongly disagree.**

**2. disagree.**

**3. neither agree or disagree (neutral).**

**4. agree.**

**5. strongly agree.**

Please circle your answer

**The workshop has...**

|  |  | 1 (strongly disagree) to 5 (strongly agree) |
| --- | --- | --- |
| 1 | Improved my knowledge on the importance of physical activity for my patients | 1 2 3 4 5 |
| 2 | Improved my knowledge of what to say to my patients about physical activity | 1 2 3 4 5 |
| 3 | Given me the skills to have a conversation about physical activity | 1 2 3 4 5 |
| 4 | Given me the tools and prompts to remember to have a conversation about physical activity | 1 2 3 4 5 |
| 5 | Helped me understand how to fit the conversation in to the time I have available | 1 2 3 4 5 |
| 6 | Told me how to get the resources I need | 1 2 3 4 5 |
| 7 | Made identifying/signposting to physical activity opportunities easier | 1 2 3 4 5 |
| 8 | Given me the confidence that others are having the conversation | 1 2 3 4 5 |
| 9 | Made me feel like I should talk about physical activity as part of my role | 1 2 3 4 5 |
| 10 | Made me believe that it is the right thing to do | 1 2 3 4 5 |
| **What was most useful in the workshop?**  **What was least useful in the workshop?** | | |

**Over the next 3 months I plan to raise physical activity with my patients...**

| **0-25% of the time** | **26-50% of the time** | **51-75% of the time** | **76-100% of the time** |
| --- | --- | --- | --- |
|  |  |  |  |
